# Baseline laboratory values and metastatic burden predict survival in addition to IMDC risk in real-world renal cell carcinoma patients treated with ipilimumab-nivolumab

**DOI:** 10.2340/1651-226X.2025.44533

**Published:** 2025-10-03

**Authors:** Alaa Kheir, Berglind Johannsdottir, Alexandra Grönn-Weiss, Lisa L. Liu, Anders Ullén, Anna Laurell, Annika Håkansson, Ingrida Verbiené, Gustav Ullenhag, Rickard Carlhed, Fernanda Costa Svedman, Magnus Lindskog, Ulrika Harmenberg

**Affiliations:** a Department of Immunology, Genetics and Pathology, Uppsala University, Uppsala, Sweden; b Department of Oncology, Akademiska University Hospital, Uppsala, Sweden; c Department of Pelvic Cancer, Genitourinary Oncology, Karolinska University Hospital, Stockholm, Sweden; d Department of Oncology-Pathology, Karolinska Institute, Stockholm, Sweden; e Department of Oncology, Karlstad Central Hospital, Karlstad, Sweden; f Theme Cancer, Karolinska University Hospital, Stockholm, Sweden; g Department of Clinical Science, Intervention and Technology, Karolinska Institute, Stockholm, Sweden

**Keywords:** renal cell carcinoma, metastatic, immune checkpoint inhibitors, ipilimumab, nivolumab, biomarkers, survival rate

## Abstract

**Background and purpose:**

Clinical tools to optimally select real-world metastatic renal cell carcinoma (mRCC) patients for treatment with ipilimumab-nivolumab remain to be identified.

**Patient and methods:**

Medical records of the first 100 mRCC patients treated with ipilimumab-nivolumab at three Swedish centers were retrospectively analyzed. Data on International Metastatic Renal Cell Carcinoma Database Consortium (IMDC) risk, baseline levels of routine blood markers, and tumor burden were collected. Outcome variables were progression-free survival (PFS), overall survival (OS), and radiological response (RR) according to clinical routine imaging.

**Results:**

At a median follow-up of 22 months, 65% had progressed or died with a median PFS of 7 months and an estimated median OS of 28 months. The RR rate was 45%, including 11% complete responses (CR). 29% had progressive disease as best response. IMDC poor-risk patients had shorter mPFS (4 vs 14 months; HR [hazard ratio] 1.90; *P* = 0.009), shorter mOS (12.5 months vs not reached; HR 4.27; *P* < 0.0001), and lower CR rate (3% vs 16%, *P* = 0.06) than IMDC intermediate/favorable patients. C-reactive protein (*a*HR 2.67; *P* = 0.040), albumin (*a*HR, 2.13; *P* = 0.039), neutrophil-lymphocyte-ratio (*a*HR, 2.8; *P* = 0.009), and > 2 metastatic sites (*a*HR, 2.13; *P* = 0.024) were associated with OS after adjusting for IMDC risk. Prior nephrectomy was not (*a*HR, 0.84; *P* = 0.62). A normal C-reactive protein was associated with an increased likelihood of CR (OR 7.2; *P* = 0.017).

**Interpretation:**

Baseline blood markers and number of metastatic sites add prognostic value independently of IMDC risk in real-word mRCC patients treated with ipililmumab-nivolumab.

## Introduction

Renal cell carcinoma (RCC), ranking 16th in global cancer incidence, presents a substantial health burden with over 400,000 new cases annually [1]. Fifteen to thirty per cent of patients present with metastatic disease or relapse following treatment for localized disease, leading to significant morbidity and mortality [2, 3].

The prognosis of patients diagnosed with metastatic renal cell carcinoma (mRCC) is stratified according to the international Metastatic Renal Cell Carcinoma Database Consortium (IMDC), classifying individuals into categories of favorable, intermediate, or poor prognosis. The determination of prognostic categories relies on the evaluation of well-established and validated patient and laboratory factors. The IMDC criteria were originally developed for patients treated with tyrosine kinase inhibitors (TKIs) [4]. Historically, the efficacy of TKI has been limited, typically producing partial responses or disease stabilization [5].

T-cell immune checkpoint inhibitors (ICIs), targeting programmed cell death protein 1/programmed death-ligand 1 (PD-1/PD-L1) or cytotoxic T-lymphocyte antigen 4 (CTLA-4), have significantly improved survival in various cancer types, including mRCC, malignant melanoma, lung cancer, and gastrointestinal cancers [6–8]. The phase III randomized Checkmate 214 trial demonstrated a significant increase in overall survival (OS) for clear cell mRCC patients with IMDC intermediate or poor risk treated with the combination of ipilimumab plus nivolumab (IPI-NIVO) versus sunitinib [9]. These results have led to the implementation of IPI-NIVO as the first-line standard therapy of non-favorable clear cell mRCC patients in 2018. In addition, the Checkmate 920 and SUNNIFORECAST trials indicated promising results using IPI-NIVO also in non-clear cell RCC [10, 11]. Recently, regimens combining ICI and TKIs have gained approval as first-line strategies in mRCC as alternatives to IPI-NIVO, though treatment choice remains challenging [12–15].

Despite that ICI treatment carries substantial risk of immune-mediated toxicity that may be severe or even detrimental, as of yet, there is no clinically useful predictive factor identified for IPI-NIVO treatment in mRCC except for sarcomatoid features present in a small subset of patients [16].

Inflammatory responses are frequent in advanced RCC, though the added value of routine laboratory tests of inflammatory activity remains unclear for ICI treatment. C-reactive protein (CRP) has shown significant prognostic correlation with RCC-specific mortality and all-cause mortality following surgical resection of localized disease [17]. In the metastatic setting, the CRP to albumin ratio, neutrophil-lymphocyte ratio (NLR), and platelet-lymphocyte ratio have demonstrated prognostic value in patients receiving sunitinib or pazopanib [18, 19]. NLR predicted longer survival in patients treated with PD-1/PD-L1 inhibitors following TKI progression [20, 21].

Lactate dehydrogenase (LDH) has also been identified as a prognostic marker, with elevated levels correlating with poor OS in mRCC patients treated with TKIs [22]. Serum albumin levels have been associated with OS outcomes in patients with RCC treated with TKIs or ICI [23, 24].

While randomized controlled trials are of fundamental value to oncology, their constraints, including stringent eligibility criteria and limited patient heterogeneity, underscore the need for real-world evidence (RWE) in this rapidly advancing era of mRCC therapy [25, 26].

In the present study, we retrospectively analyzed the first 100 patients with mRCC treated with ipilimumab-nivolumab in east-mid Sweden. Our aims were (1) to study if IMDC risk categories remain prognostic in this treatment setting and (2) to analyze if baseline laboratory or clinical parameters, other than those included in the IMDC standard risk algorithm, contribute to prognostic information.

## Patients and methods

### Study design and patient population

This was a retrospective, multicenter study. Three hospitals in Mid Sweden recruited patients: Karolinska University Hospital, Uppsala University Hospital, and Central Hospital in Karlstad. Since the approval by Swedish authorities in March 2019, the first 100 patients treated with IPI-NIVO were analyzed with data cut-off from April 28, 2023. Relevant data extracted from medical records included age, gender, tumor histopathology (diagnosis, ISUP grade, sarcomatoid features), date of primary and metastatic diagnosis, metachronous or synchronous metastatic disease, prior cytoreductive nephrectomy, number and type of organs involved with metastatic disease at treatment start, and treatment details.

IPI-NIVO was administered according to clinical routine, i.e., with the aim to give four courses of doublet treatment with IPI (1 mg/kg) and NIVO (3 mg/kg) every 3 weeks, followed by radiological and clinical evaluation. With acceptable tolerance and radiological response (RR) or stable disease (SD) at computed tomography (CT) evaluation after 3 months, patients continued maintenance treatment with NIVO monthly or every other week. The treatment continued until disease progression or unacceptable toxicity or could be discontinued after 2 years, provided maintained disease control. Data on treatment cycles were retrieved as well as date and cause of ICI treatment discontinuation, and details on post-progression treatment.

Eastern Cooperative Oncology Group (ECOG) performance status was used as an estimate of patient’s functional level, defined at the visit to the oncology department closest in time prior to start of IPI-NIVO. ECOG was preferred over Karnofsky index (KPS) due to its more abundant use in everyday clinical practice in Sweden. An ECOG of 2 or more was considered equivalent to KPS < 80%. The IMDC risk prior to treatment was calculated for each patient. Variables contributing to IMDC risk score included hemoglobin level < lower limit of normal (1 point), platelet level > upper limit of normal (1 point), neutrophil count > upper limit of normal (1 point), albumin-corrected calcium > upper limit of normal (1 point), ECOG ≥ 2 (1 point), and time from diagnosis of primary RCC to systemic treatment < 1 year (1 point). For prognostic analysis, patients with IMDC favorable risk (0 risk factors) and intermediate risk (1–2 risk factors) were grouped together (Int/Fav) and compared to IMDC poor-risk patients (Poor) (≥ 3 risk factors).

In addition to lab parameters necessary for IMDC risk score calculation, baseline levels of the following blood markers were obtained and tested for prognostic relevance: albumin (cut-off, 34 g/L), CRP (cut-off, 10 mg/L), LDH (cut-off, upper limit of normal), and neutrophil-to-lymphocyte ratio (cut-off, 4). All laboratory data were dichotomized (Table 1). Prior cytoreductive nephrectomy and number of metastatic sites were considered indicators of tumor burden.

**Table 1:** Demographic and clinical characteristics at baseline.

Characteristic	Patients
Study population – no. (%)	100 (100)
Median age (range ) – yr	65 (41–80)
Sex – %	
Male	68
Female	32
ECOG performance status – %	
0	47
1	34
2	16
3	3
IMDC prognostic risk group – %	
Favorable	7
Intermediate	55
Poor	38
M1 – %	73
Previous nephrectomy – %	78
Histology – %	
Clear cell	90
Papillary	4
Chromophobe	1
Collecting duct carcinoma	1
Other or unclassifiable	4
Sarcomatoid features – %	19
Number of metastatic organs or sites – %	
1	22
2	50
> 2	28
Sites of metastasis – %	
Lung	77
Lymph nodes	54
Bone	31
Adrenal	13
Liver	12
Brain	3
Anemia – %	48
Platelets elevated – %	31
Hypercalcemia* – %	35
Neutrophils elevated – %	29
LDH elevated – %	28
CRP > 10 mg/L – %	60
Albumin < 34 g/L – %	32

* Albumin-corrected. ECOG: Eastern Cooperative Oncology Group performance status; IMDC: International Metastatic Renal Cell Carcinoma Database Consortium; M1: synchronous metastases at primary diagnosis; LDH: plasma Lactate Dehydrogenase; CRP: plasma C-reactive protein.

Endpoints included progression-free survival (PFS), OS, and best RR according to clinical routine imaging. PFS was defined as time from start date of IPI-NIVO to radiological progression or death, whichever occurred first. OS was defined as time from start date of IPI-NIVO until death or last follow-up. Progression was defined radiologically based on standard clinical evaluation and not on RECIST criteria. A partial response (PR) was defined as any tumor shrinkage, whereas lack of meaningful difference in any lesion was defined as SD. Progressive disease (PD) was defined as appearance of new tumor lesions or unequivocal increase in size of any tumor lesion compared to baseline. Radiological and clinical follow-up during and following IPI-NIVO typically included CT of chest and abdomen every 3 months. Patients were evaluated clinically prior to each doublet cycle, prior to NIVO maintenance and thereafter at least every 3 months.

### Statistical analysis

Kaplan–Meier curves were constructed, and Cox proportional hazards were calculated. Hazard ratios (HRs) with 95% confidence intervals (CI) were calculated by univariate and multivariate analyses. The chi-squared test was used to test associations with best RR. *P* < 0.05 was considered significant. STATISTICA version 13 (StatSoft Inc, Tulsa, USA) was used.

### Ethics

This study was approved by the Swedish Ethical Review Authority (Dnr 2023-03640-01).

## Results

### Patients and treatments

Baseline demographics and disease characteristics are described in Table 1. Histology was predominantly clear cell. Seventy-three per cent had synchronous metastases, and 78% had prior nephrectomy. Nineteen per cent had an ECOG performance status of ≥2. Median age was 65 years. Two-thirds were male. Most patients had IMDC Int (55%) or poor risk (38%), with Fav risk in seven patients (7%). Twenty-eight per cent had metastatic involvement of more than two organs. Lungs, lymph nodes, and bone were the most involved organs (Table 1). Ninety-five per cent received IPI-NIVO in first line, four in second line (following progression on antiangiogenic-targeted agent), and one patient in third line.

Patients with the primary in place were more likely to have an elevated CRP (OR 16.2; 95% CI 3.56–73.76; *p* = 0.0003) or a decreased albumin (OR 2.57; 95% CI 1.04–6.36; *p* = 0.040) compared to patients with prior nephrectomy. Similar associations were seen for patients with > 2 metastatic sites (OR 2.88 for elevated CRP; 95% CI 1.03–8.06; *p* = 0.044; and OR 6.0 for decreased albumin; 95% CI 2.26–15.82; *p* = 0.0003). In addition, patients with < 12 months from primary RCC diagnosis to initiation of ipilimumab-nivolumab were more likely to have an elevated CRP compared to patients who initiated treatment later (OR 3.71; 95% CI 1.32–10.47; *p* = 0.013). No associations were found for the other blood markers (data not shown).

Median follow-up of alive patients was 22 months. At cut-off, ICI treatment was ongoing in 17% of patients. The primary reason for ICI discontinuation was disease progression (41%), followed by toxicity (35%). Patients discontinuing due to toxicity had a median treatment duration of 2.0 months (range 0.3–14.7). Two patients suffered fatal toxicity due to myocarditis (after 4.1 months) and liver failure (after 1.1 months). Six patients with objective response discontinued treatment, either according to plan or at own will, after a median duration of 25 months (range 13–36 months). Among patients who discontinued ICI, 53% received subsequent systemic therapy with cabozantinib (39%), sunitinib (5%), axitinib (4%), or pazopanib (2%). One patient received bevacizumab, everolimus, and nivolumab-cabozantinib as post-progression therapy.

### Efficacy

#### Treatment response

Radiologic response data were available for 93 patients. Median time to best response was 2.6 months (range 1.1–14.2). The response rate was 45%, including 11% complete response (CR) and 34% PR (Table S1). Median duration of response was 12.9 months (0.6 to not reached). Nineteen per cent had SD, while 29% had PD as a best response. PD was numerically more common in IMDC Poor-risk patients compared to Int/Fav risk (39.5% vs 22.5%; odds ratio 2.2; 95% CI 0.93–5.40; *P* = 0.07). There was a trend for higher CR rate in IMDC intermediate/favorable patients than poor-risk patients (16% vs 3%; *P* = 0.06). All 11 patients with CR had ongoing response and were alive at data cut-off after median follow-up of 32.3 months (range 7.3–44.0), while 41% of partial responses were ongoing. Factors associated with CR are shown in Table S2. Patients with lung or mediastinal lymph node metastases only were more likely to achieve a radiologic response compared to patients with other metastatic sites (70% vs 42%; odds ratio 3.2; 95% CI 1.09–9.15; *p* = 0.033), with borderline significance for CR (20% vs 8%; odds ratio 3.7; 95% CI 1.00–13.83; *p* = 0.050). Liver or bone metastases were not associated with radiologic response (*p* = 0.40 and *p* = 0.48) or with CR (Table S2).

#### Progression-free and overall survival

**Figure 1:**
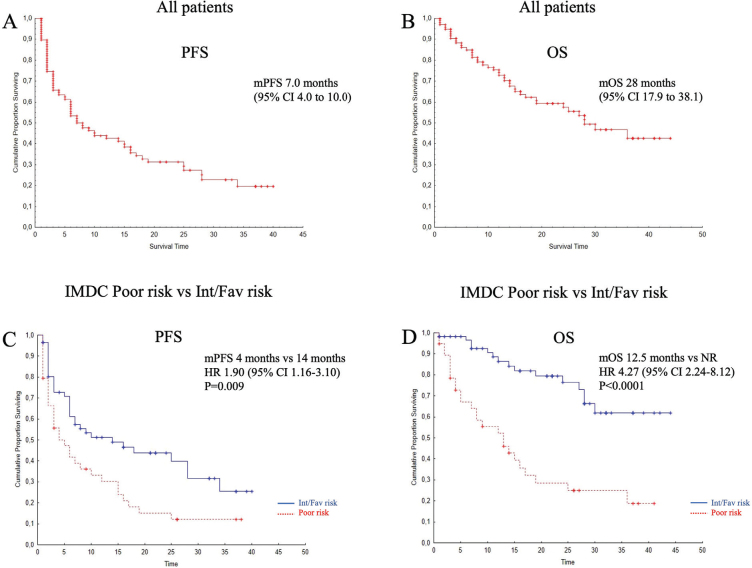
Kaplan–Meier curves for progression-free survival (PFS) and overall survival (OS) in all patients (A–B) and stratified by IMDC risk groups (C–D). IMDC: International Metastatic Database Consortium; Int/Fav: IMDC intermediate or favorable risk; NR: Not Reached; HR: hazard ratio; CI: confidence interval.

Sixty-five per cent of patients had progressed or died at data cut-off. Median PFS was 7.0 months [95% CI 4.0–10.0], and IMDC Poor-risk patients had significantly shorter PFS than intermediate or favorable risk (median 4 vs 14 months; HR 1.90; 95% CI 1.16–3.10; *p* = 0.009) (Figure 1).

At data cut-off, 41% had died. Estimated median OS was 28 months [95% CI 17.9–38.1]. IMDC Poor patients had significantly shorter OS than Int/Fav patients (median 12.5 months vs not reached; HR 4.27; 95% CI 2.24–8.12; *p* < 0.0001; Figure 1).

**Figure 2:**
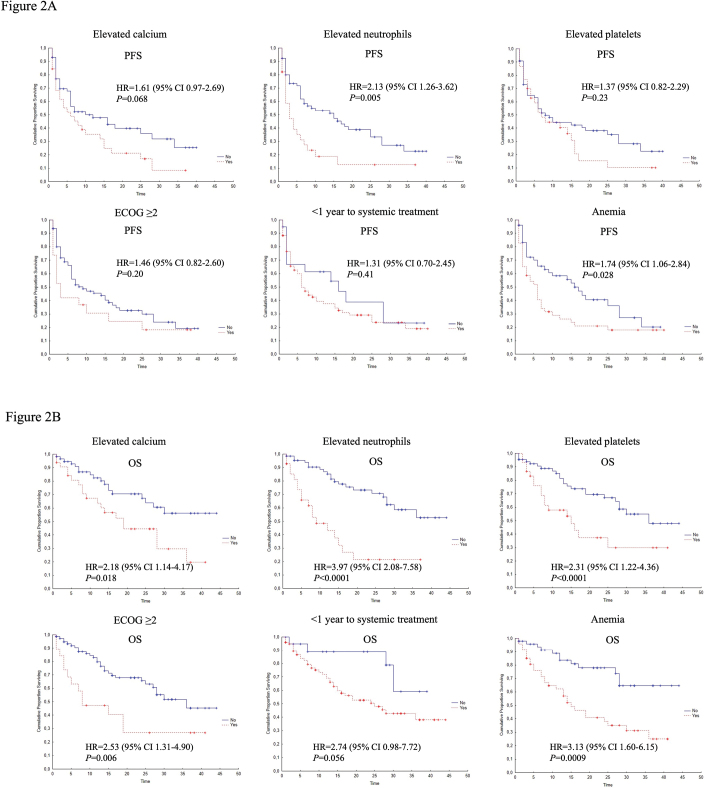
Kaplan–Meier curves for PFS (A) and OS (B) in relation to individual IMDC risk factors: Elevated Calcium, Elevated Neutrophils, Elevated Platelets, ECOG ≥ 2, < 1 year to start of systemic treatment, Anemia. PFS: progression-free survival; OS: overall survival; HR: hazard ratio; CI: confidence interval; ECOG: Eastern Cooperative Oncology Group.

**Figure 3:**
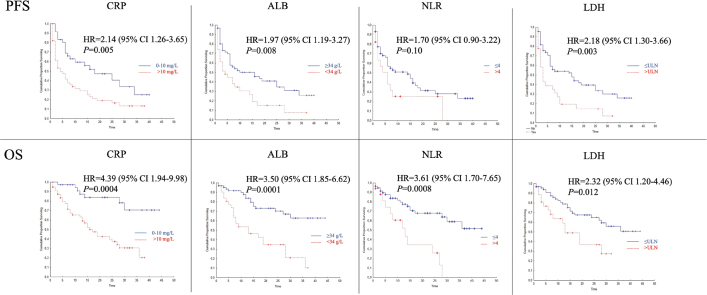
Associations between inflammatory, immune cell composition, and disease activity markers and survival. Upper panels indicate progression-free survival (PFS) and lower panels overall survival (OS). C-reactive protein > 10 vs ≤ 10 mg/L (CRP), serum albumin < 34 vs ≥ 34 g/L (ALB), neutrophil-to-lymphocyte ratio > 4 vs ≤ 4 (NLR), and lactate dehydrogenase elevated vs normal (LDH). HR: hazard ratio; CI: confidence interval.

Of the six IMDC risk factors, neutrophilia (HR 2.13; 95% CI 1.26–3.62; *p* = 0.005) and anemia (HR 1.74; 95% CI 1.06–2.84; *p* = 0.028) were associated with shorter PFS (Figure 2A). All factors but time from diagnosis to systemic treatment were associated with OS, with pretreatment neutrophilia discriminating best (Figure 2B). Among additional blood markers analyzed, CRP (*n* = 95), albumin (*n* = 97), NLR (*n* = 78), and LDH (*n* = 97) were associated with OS and, with the exception of NLR, with PFS in unadjusted analysis (Figure 3).

**Figure 4:**
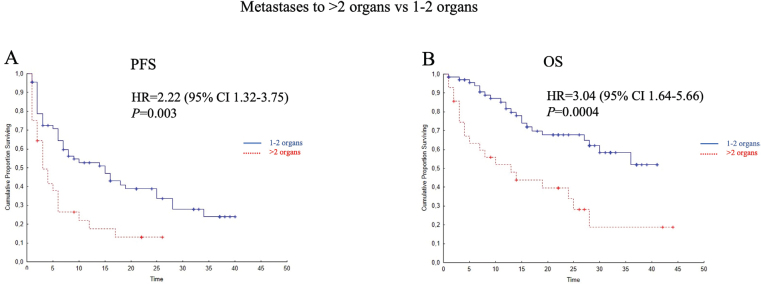
Kaplan–Meier curves illustrating associations of metastatic burden (> 2 organs with metastases vs 1–2 organs involved) with progression-free (PFS; A) and overall survival (OS; B). HR: hazard ratio; CI: confidence interval.

Patients with metastases in > 2 organs had shorter PFS and OS than those with the involvement of 1–2 organs (Figure 4). Prior nephrectomy did not affect PFS but was associated with longer OS in unadjusted analysis (Table 2).

**Table 2:** Prognostic factors in patients treated with ipilimumab-nivolumab, crude, and adjusted for IMDC risk group.

Variable	HR Progression/death crude	95% CI	*P*	HR Progression/death adjusted for IMDC	95% CI	*P*	HR Death crude	95% CI	*P*	HR Death adjusted for IMDC	95% CI	*P*
IMDC Poor	1.90	1.16–3.10	0.010	NA	NA	NA	4.27	2.24–8.12	< 0.0001	NA	NA	NA
LDH	2.18	1.30–3.66	0.003	2.01	1.19–3.40	0.009	2.32	1.20–4.46	0.012	1.80	0.92–3.52	0.086
ALB	1.97	1.19–3.27	0.008	1.64	0.94–2.86	0.085	3.50	1.85–6.62	0.0001	2.13	1.04–4.37	0.039
NLR	1.70	0.90–3.22	0.10	1.54	0.80–2.96	0.20	3.61	1.70–7.65	0.0008	2.81	1.29–6.14	0.009
CRP	2.14	1.26–3.65	0.005	1.94	1.01–3.70	0.045	4.39	1.94–9.98	0.0004	2.67	1.05–6.82	0.040
> 2 Org	2.22	1.32–3.75	0.003	1.88	1.08–3.28	0.025	3.04	1.64–5.66	0.0004	2.13	1.11–4.10	0.024
Nephrectomy	0.67	0.40–1.12	0.13	0.94	0.51–1.70	0.83	0.43	0.23–0.81	0.0085	0.84	0.42–1.68	0.62

HR: hazard ratio; CI: confidence interval; IMDC Poor: International Metastatic RCC Database Consortium Poor-risk group; Crude: unadjusted for IMDC risk group; Adjusted: Adjusted for IMDC risk group; LDH: lactate dehydrogenase higher than upper normal limit at baseline; ALB: albumin < 34 g/L at baseline; CRP: C-reactive protein > 10 mg/L at baseline; NLR: Neutrophil-to-lymphocyte ratio greater than 4 at baseline; > 2 Org: metastases in more than organ systems at baseline; Nephrectomy: prior nephrectomy.

Liver metastases did not affect PFS (*P* = 0.17) but were associated with worse OS (median 12.5 vs 36 months; HR 2.26; 95% CI 1.04–4.92; *P* = 0.039). Bone metastases were not associated with PFS (*P* = 0.78) or OS (*P* = 0.17). There were no significant differences in PFS (*p* = 0.13) or OS (*p* = 0.14) when comparing patients with lung and lymph node metastases only against patients with other metastatic sites.

Information on sarcomatoid features was available for 86 patients. Sarcomatoid features were present in 19 patients (22%) but not associated with PFS or OS (data not shown).

Patients achieving RR had longer OS than those without a response (median not reached vs 19 months; HR 0.40; 95% CI 0.21–0.79; *P* = 0.008).

Patients with non-clear cell RCC (*n* = 10) did not differ from clear cell patients in terms of median OS (27 months vs 29 months, *p* = 0.63), median PFS (8 months vs 7 months, *p* = 0.45), or the radiologic response rate (50% vs 44%, OR 1.25, 95% CI 0.34–4.62; *p* = 0.74). CR was numerically more common in non-clear cell patients (2 out of 10 patients, 20%) compared with clear cell patients (9 out of 83 patients with known response data, 11%), but the difference was not statistically significant (OR 2.01; 95% CI 0.38–11.22; *p* = 0.41).

#### Survival adjusted for IMDC risk

In addition to unadjusted analyses, Table 2 shows hazard ratios for the respective blood markers and tumor burden indicators following adjustment for IMDC risk (Poor vs Int/Fav). All blood markers but LDH remained significantly associated with OS when adjusting for IMDC risk. With respect to tumor burden, number of metastatic sites > 2 remained associated with OS when adjusted for IMDC risk, whereas prior nephrectomy lost its association with OS when adjusting for IMDC risk (Table 2).

### Added value of baseline markers within the respective IMDC groups

We further performed exploratory analyses, separately for each risk group, to investigate if any of the baseline factors could enhance the power of IMDC predictivity. In IMDC Int/Fav patients, signs of systemic inflammation (elevated CRP or low serum albumin) were associated with shorter PFS and shorter OS as compared to normal levels of CRP or albumin. Only two patients with IMDC Poor-risk had normal CRP, precluding further testing within this subgroup. Albumin level seemed to add little prognostic information in the IMDC Poor-risk group (Figure S1).

The adverse effect of an elevated LDH on PFS was evident in both the IMDC Int/Fav and IMDC Poor-risk groups. The effect was less pronounced with respect to OS in both risk groups (Figure S1).

Most IMDC Int/Fav risk patients had 1–2 metastatic sites, limiting the power to detect a possible added prognostic effect of metastatic burden in these patients. However, in IMDC Poor-risk patients, the involvement of > 2 metastatic sites was associated with a particularly poor PFS and OS (Figure S1).

## Discussion and conclusion

Optimal patient selection for IPI-NIVO remains a challenge. This study reports outcomes of the first 100 mRCC patients treated with IPI-NIVO in a real-world setting across three Swedish centers. Our results confirm the relevance of the IMDC prognostic score and identify additional pretreatment blood markers as independent prognostic factors. We also show that the number of metastatic sites, as opposed to prior nephrectomy, is associated with survival following IPI-NIVO. While IPI-NIVO is primarily approved for intermediate and high-risk patients, data may suggest its value for patients with favorable risks with double CR rate and more durable response [27]. In our cohort, seven patients had favorable risk, which may reflect evolving clinical practices toward application of this combination.

The IMDC criteria were initially developed to predict survival in RCC patients treated with TKIs. In the Checkmate-214 trial, patients with IMDC risk factors receiving IPI-NIVO showed superior survival and higher response rates than those on sunitinib. Our finding that IMDC poor-risk patients treated with IPI-NIVO outside of trials fare worse than intermediate or favorable-risk patients is consistent with a recent multinational chart study of real-world mRCC patients [25]. As in that study, we found that the impact of IMDC risk group was greater on OS than PFS. Five of the six IMDC risk factors were significantly associated with OS in our cohort. Time to treatment did not reach significance, likely due to the small number of patients treated > 1 year from diagnosis. Neutrophil counts and hemoglobin level were the only IMDC factors significantly associated with PFS.

Beyond IMDC factors, we evaluated additional baseline blood markers including CRP (inflammation), albumin (inflammation and/or nutritional status), NLR (immune cell balance), and LDH (putative marker of disease activity). CRP and albumin remained prognostic when adjusting for IMDC risk. A previous study of 74 IPI-NIVO-treated patients linked CRP to OS but did not adjust for IMDC risk [28]. Another retrospective study of early CRP kinetics in first-line ICI-treated mRCC patients found normalization within 3 months to be associated with better survival, independently of IMDC risk [29]. Pretreatment CRP as a continuous variable was also linked to OS and PFS, independently of IMDC risk in patients receiving nivolumab monotherapy [30]. Biomarkers may differ between PD-1 inhibitor monotherapy and combined PD-1 and CTLA4 inhibition [31]. Our findings reinforce the prognostic utility of baseline CRP in IPI-NIVO-treated mRCC. A cut-off of 10 g/L was found to be useful; lower or higher thresholds (< 5 or > 50 g/L) did not improve discrimination (data not shown). These findings align with Tachibana et al. who, in a smaller study, found an association of normal baseline CRP associated with longer PFS and higher likelihood of response, although OS was not assessed [32]. In contrast, Schuttke et al. reported no association of CRP with PFS or OS; however, 16 of 61 patients received IPI-NIVO (*n* = 61) [33]. No data were available on subsequent CRP levels during treatment in our study, which potentially may be valuable for early treatment monitoring as previously suggested [29, 32–34].

We and others have previously reported pretreatment albumin to be prognostic in TKI-treated mRCC patients [24]. Here, we confirm albumin to be associated with survival after IPI-NIVO treatment. This aligns with findings in melanoma and supports the broader association of albumin with survival in ICI-treated patients found in the US Oncology Network study [23, 35]. Ekinci et al. also identified albumin as prognostic in mRCC patients treated with single agent nivolumab [36].

Since NLR reflects the peripheral blood immune cell balance, it may have relevance in the context of immunotherapy. A meta-analysis reported associations of unadjusted NLR with OS and PFS using different cut-offs [37]. In the present study, we used NLR > 4 and found it to be significantly associated with OS, and importantly to remain prognostic after adjusting for IMDC risk [38].

Elevated LDH, seen in 10–15% of mRCC cases, indicates poor prognosis and was part of the earlier used MSKCC prognostic criteria [39]. The IMDC criteria, which excludes LDH, were, however, found to perform better in predicting survival in the targeted therapy era [4]. When adjusting for IMDC risk, we found LDH to predict PFS but not OS. Expectedly, therefore, we observed a strong association of elevated LDH with other adverse prognostic factors included in the IMDC risk algorithm (data not shown).

We also explored associations of tumor burden with survival following IPI-NIVO. Prior nephrectomy was associated with OS in crude analysis but not after adjusting for IMDC risk group. No PFS association was seen. In a large observational study, upfront nephrectomy was associated with improved OS in ICI- and TKI-treated patients, although those with adverse risk features such as poor performance status, bone, brain or liver metastasis, or IMDC poor risk were least likely to be treated with upfront nephrectomy [40]. There was a significantly better OS after nephrectomy in patients treated with either ICI or TKI. Randomized data on nephrectomy in the ICI era are still lacking; several studies are ongoing (NORDIC SUN NCT 03977571, PROBE NCT 04510597, and CYTO-KIK NCT 04322955).

With respect to metastatic involvement, we found > 2 involved organs to be prognostic. In contrast, 1- vs > 1-involved organ was not associated with PFS or OS nor were higher cut-off values than 2-involved organs (data not shown). These findings are consistent with Checkmate-025, which showed worse OS in patients with ≥2 metastatic sites on nivolumab monotherapy [41]. Of potential interest, we found a significant association with radiologic response rate in favor of patients with lung or mediastinal lymph node involvement only compared to other metastatic sites. This, however, did not translate into any PFS or OS benefit in our cohort. Nevertheless, our data suggest that it could be of interest to undertake future investigations into the immune landscape of metastatic RCC limited to lungs and the mediastinum.

We found associations between both inflammatory markers (CRP and albumin) with tumor burden and time to treatment initiation. However, in multivariate analysis, controlling for nephrectomy status, number of metastatic sites, and IMDC risk group, CRP remained independently associated with PFS, and both CRP and albumin remained independently associated with OS. Therefore, they were not merely surrogates of a high tumor burden or disease pace but contributed with independent prognostic information.

We undertook exploratory analyses to investigate if any of the baseline factors could enhance the power of IMDC predictivity. Our data indicate that IMDC Int/Fav risk patients with systemic inflammation (elevated CRP or low serum albumin) had relatively similar PFS (in median 3–5 months) to that of IMDC Poor-risk patients (in median 4 months). This implies a risk migration in the presence of inflammatory parameters, which could be of potential use when predicting the outcome with ipilimumab-nivolumab in individuals with intermediate of favorable risk according to the IMDC. The combination of IMDC Poor risk with either elevated LDH or with > 2 metastatic sites predicted a particularly grave course of the disease in our cohort. These findings may refine the IMDC risk stratification, though further prospective validation is needed.

Sarcomatoid features were not associated with response or survival in our cohort. In the IMDC analysis by Santoni et al., sarcomatoid features were associated with worse survival in intermediate-risk patients but offered no additional value in poor-risk cases [25]. In our cohort, the number of patients with sarcomatoid features was too small for subgroup analysis.

Our study included 10% non-clear cell RCC patients, reflecting real-world diversity. Overall, the findings for these patients did not differ meaningfully in terms of likelihood of an RR or with respect to PFS or OS. This is in line with evidence of IPI-NIVO efficacy across histology types [10, 11]. CR were seen in two non-clear cell patients: one with a MiT family translocation cell carcinoma and one patient with a papillary type 2 RCC.

In this real-world cohort, five patients received IPI-NIVO in later treatment lines. This contrasts with the approval in first-line setting but likely reflects clinical practice. Two partial responses were seen among these patients.

Limitations of this study include its retrospective design, modest cohort size, lack of RECIST-based response classification, and relatively limited follow-up time. We assessed tumor response per clinical routine, not according to RECIST. As a consequence, the proportion of patients with PD as best response could have been overestimated, since formally using RECIST, PD would necessitate > 20% increase in the sum of tumor diameters or a novel lesion appearing, while clinical routine assessment might have defined also smaller increases in tumor burden as PD. Conversely, the proportion of patients with PR might be overestimated due to misclassification of patients that would be considered SD if RECIST was used. With this limitation in mind, we focused our analysis on the CR rate (disappearance of all metastatic lesions). Factors associated with CR included an ECOG of 0, IMDC intermediate or favorable risk, a normal pretreatment CRP, and absence of anemia. We found strikingly similar CR rates in this real-world cohort as compared to what was reported for trial subjects in the Check Mate 214 study [9]. This is reassuring and indicates that IPI-NIVO is, indeed, a very active regimen also in real-world mRCC patients.

Although the optimal first-line regimen remains unclear, the ongoing CARE-1 trial (NCT06364631), which randomizes patients between IPI-NIVO and IO-TKI regimens, may offer critical insights into choosing the optimal first-line treatment in mRCC.

In conclusion, we show that specific pretreatment blood markers and metastatic burden provide prognostic information in addition to IMDC risk classification in mRCC patients receiving IPI-NIVO. Larger, prospective studies are needed to confirm these findings before integrating such markers into routine clinical treatment selection.

## Supplementary Material





## Data Availability

The data supporting the findings of this study are derived from retrospective medical records of mRCC patients. Due to the sensitive nature of individual patient health information, even though all data have been anonymized, the underlying dataset cannot be made publicly available. The anonymized data will be securely stored on institutional servers at Uppsala University Hospital for a minimum of 10 years in accordance with national regulations and institutional policies. Researchers wishing to access the data for academic purposes may submit a request to the corresponding author to ensure compliance with confidentiality requirements.
