# Supplementary material for: Baseline laboratory values and metastatic burden predict survival in addition to IMDC risk in real-world renal cell carcinoma patients treated with ipilimumab-nivolumab
Source: Acta Oncol. 2025 Oct 3;64:44533. doi: 10.2340/1651-226X.2025.44533 (PMC12509422; doi:10.2340/1651-226X.2025.44533)

Supplementary material has been published as submitted. It has not been copyedited, or typeset by Acta Oncologica

*Figure S1:* Progression-free survival and Overall survival by different IMDC risk groups and baseline biomarker status. C-reactive protein (A-B), Albumin (C-D), Lactate dehydrogenase (E-F), Metastatic burden (G-H)

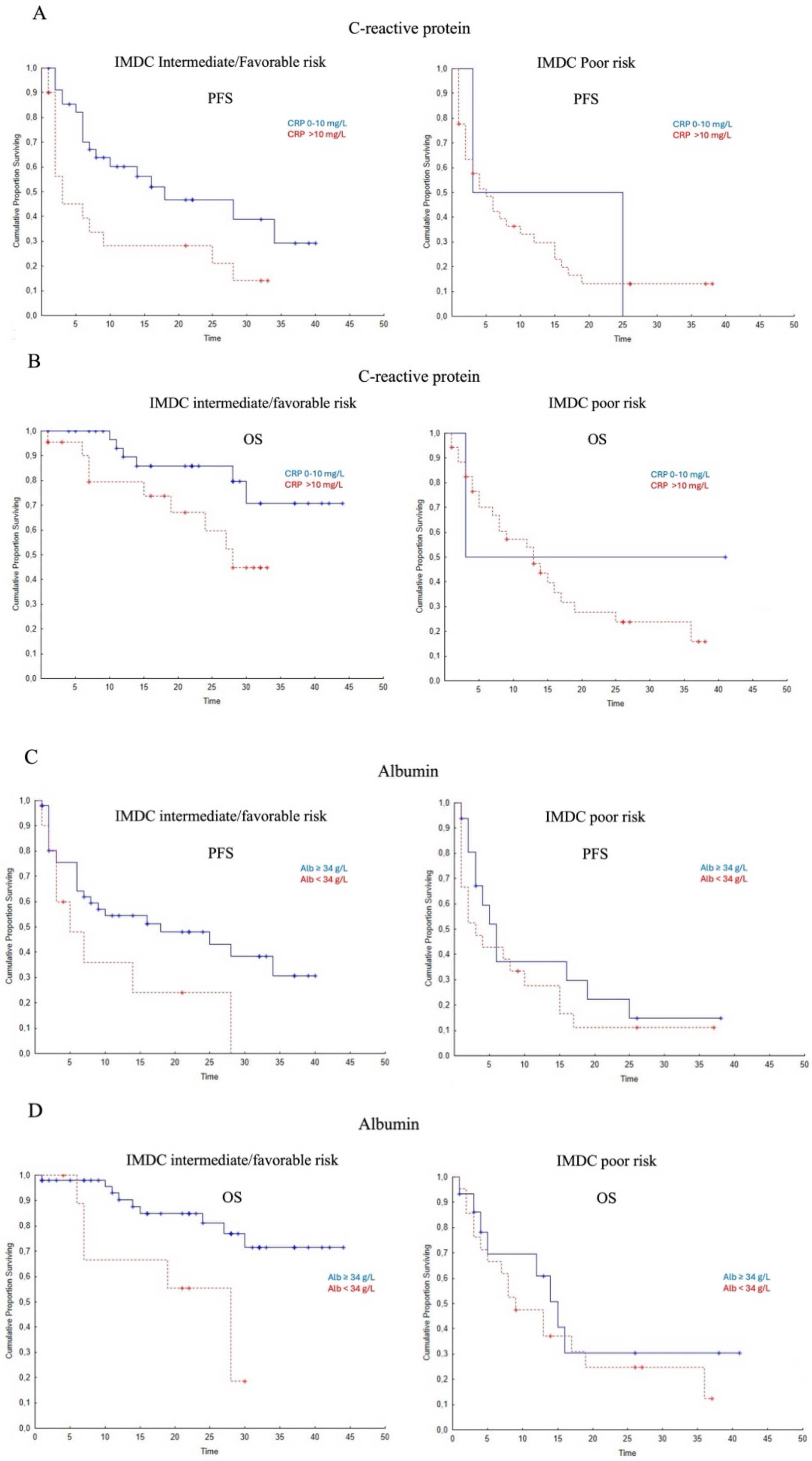

E

Lactate dehydrogenase

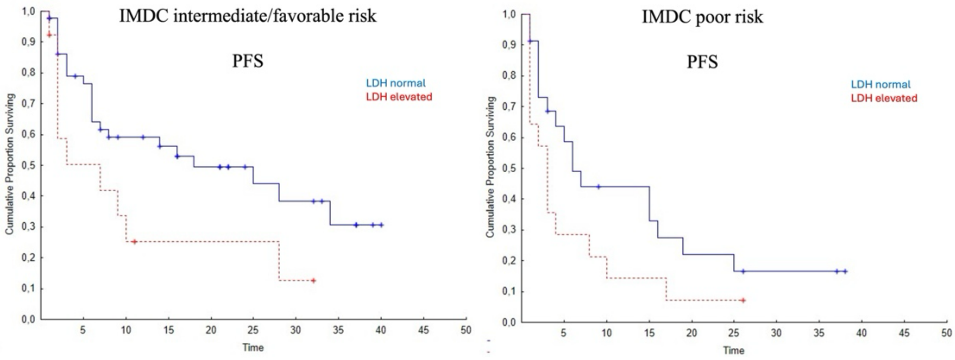


F

Lactate dehydrogenase

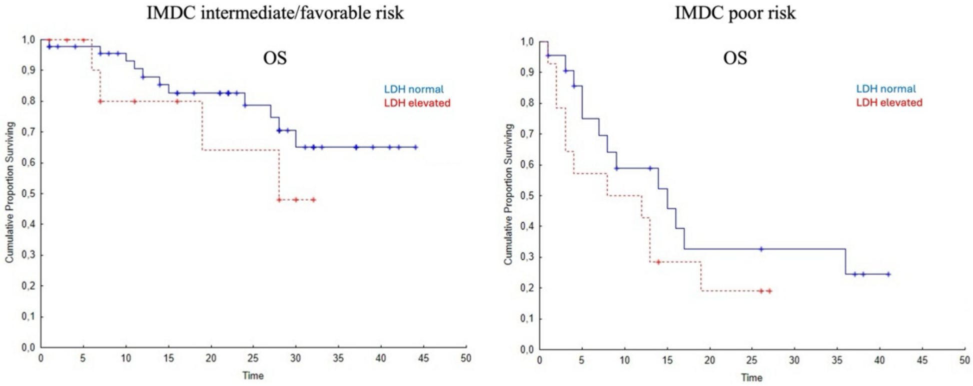


G

Metastatic burden

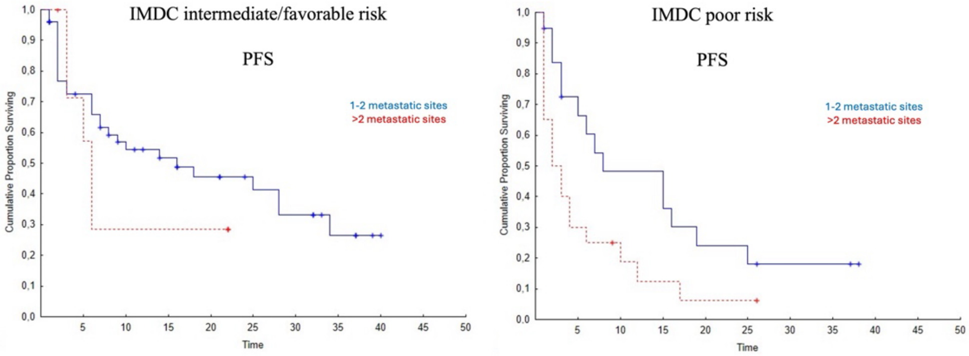


H

Metastatic burden

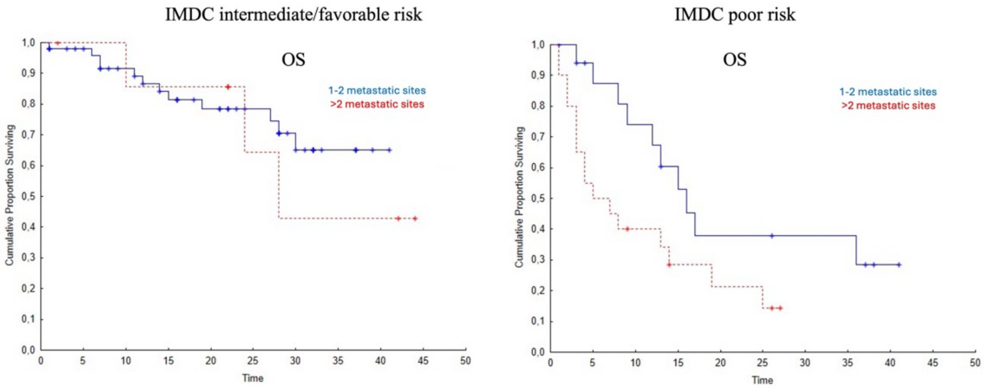

Supplement: Supplementary file 1 [file AO-64-44533-s1.pdf]
