# Supplementary material for: Baseline laboratory values and metastatic burden predict survival in addition to IMDC risk in real-world renal cell carcinoma patients treated with ipilimumab-nivolumab
Source: Acta Oncol. 2025 Oct 3;64:44533. doi: 10.2340/1651-226X.2025.44533 (PMC12509422; doi:10.2340/1651-226X.2025.44533)
Supplement: Supplementary file 2 [file AO-64-44533-s2.pdf]

Supplementary material has been published as submitted. It has not been copyedited, or typeset by Acta Oncologica

Table S1: Best radiological response* to ipilimumab-nivolumab.

Measure	ALL patients (N=100)	IMDC Int/Fav risk (n=62)	IMDC Poor risk (n=38)
Best overall response – no. (%)			
Complete response	11 (11)	10 (16)	1 (3)
Partial response	34 (34)	19 (31)	16 (42)
Stable Disease	19 (19)	15 (24)	4 (10.5)
Progressive disease	29 (29)	14 (22.5)	15 (39.5)
Unknown	7 (7)	4 (6.5)	2 (5)
Disease control rate	64 (64)	41 (66)	21 (55)
Median time to response (range) – months	2.6 (1.1–14.2)	2.7 (1.1–10.5)	2.4 (0.7–14.7)
Median duration of response (range) – months	12.9 (0.6 – not reached)	13.8 (1.5 – not reached)	12.4 (0.6 – not reached)

*According to clinical routine evaluation with computed tomography. IMDC =International Metastatic RCC Database Consortium risk group; Fav=favorable risk, Int=intermediate risk.

Table S2: Pre-treatment variables and the likelihood for complete response to ipilimumab-nivolumab.

**Variable**	Odds ratio (95% CI) **complete response***	P
IMDC Int/Fav vs Poor	7.3 (0.9–59.6)	0.064
ECOG		
0 vs 1-2	6.2 (1.3–30.6)	0.025
0-1 vs ≥2	2.4 (0.3–20.3)	0.41
Neutrophils		
normal vs elevated	4.5 (0.6–37.5)	0.16
Hemoglobin		
normal vs low	5.2 (1.1–25.6)	0.04
Calcium*		
normal vs elevated	2.4 (0.5–12.2)	0.28
Platelets		
normal vs elevated	2.3 (0.5–11.5)	0.30
Time to treatment		
≥1 year vs < 1year	1.4 (0.3–6.0)	0.62
Prior nephrectomy		
yes vs no	12.0 (0.7–211.6)	0.09
Metastatic sites		
1-2 vs >2	4.4 (0.5–36.1)	0.17
Lung or mLN met	3.7 (1.0–13.8)	0.05
Liver met	0.7 (0.1–6.2)	0.77
Bone met	0.2 (0.0–1.7)	0.14
CRP		
normal vs elevated	7.2 (1.4–36.1)	0.017
Albumin		
normal vs low	5.9 (0.7–48.3)	0.10
LDH		
normal vs elevated	4.4 (0.5–35.4)	0.18
NLR		
≤4 vs >4	5.7 (0.3–103.6)	0.24
Sarcomatoid		
yes vs no	1.5 (0.4–6.5)	0.57

IMDC =International Metastatic RCC Database Consortium risk group; Fav=Favorable risk, Int=Intermediate risk, Poor=Poor risk; ECOG=Eastern Cooperative Oncology Group performance status; Neutrophils elevated=Neutrophil count higher than upper normal limit at baseline; PLT elevated=Platelet count >400 x10^9/L at baseline; mLN=mediastinal lymph nodes CRP=C-reactive protein >10 mg/L at baseline; Albumin low= <34 g/L at baseline; LDH=lactate dehydrogenase higher than upper normal limit at baseline; NLR=Neutrophil-to-lymphocyte ratio greater than 4 at baseline; *Albumin-corrected.
